# Identification of Disease Risk DNA Variations is Shaping the Future of Precision Health

**DOI:** 10.3390/genes10060450

**Published:** 2019-06-13

**Authors:** Walid D. Fakhouri, Ariadne Letra

**Affiliations:** 1 Center for Craniofacial Research, Department of Diagnostic and Biomedical Sciences, School of Dentistry, University of Texas Health Science Center at Houston, Houston, TX 77054, USA; Ariadne.M.Letra@uth.tmc.edu; 2 Pediatric Research Center, McGovern Medical School, University of Texas Health Science Center, Houston, TX 77030, USA

**Keywords:** genomic evolution, coding DNA variations, noncoding DNA variations, alternative transcriptional start site, alternative splicing and mRNA stability, post-transcriptional and -translational regulation

## Abstract

In recent years, the knowledge generated by decoding the human genome has allowed groundbreaking genetic research to better understand genomic architecture and heritability in healthy and disease states. The vast amount of data generated over time and yet to be generated provides the basis for translational research towards the development of preventive and therapeutic strategies for many conditions. In this special issue, we highlight the discoveries of disease-associated and protective DNA variations in common human diseases and developmental disorders.

Sequencing of the whole genome of many organisms has provided the scientific community with a tremendous amount of information to determine which part of the mammalian genome is under constraint or undergoing rapid turnover. Based on DNA conservation, evolutionary studies have shown that DNA changes are taking place at a higher rate in noncoding regulatory regions and at a much lower rate within gene coding sequences [1]. Each human cell has about 22,000 genes that are distinctly regulated over time and location to determine the fate of each cell. The code for regulating these thousands of genes is encrypted, particularly in noncoding DNA sequences and the associated epigenome modifications [1]. DNA variations in regulatory elements can disrupt gene expression and alter epigenome modifications, whereas coding mutations can alter protein function, stability, or localization [2,3]. Notably, molecular and genetic studies using animal models have shown that DNA variations play a critical role in increasing fitness to environmental conditions [4]. In contrast, certain DNA variations increase the risk for Mendelian and complex diseases [5,6,7,8]. 

To emphasize the importance of gene regulation, genome-wide association studies (GWAS) of human common diseases demonstrate that ~10% of the disease-related single nucleotide polymorphisms (SNPs) are located in amino acid coding sequences, whereas around 90% of the disease-associated SNPs fall outside of protein coding regions [2,3,9]. Identification of pathological DNA variants is critical for early diagnosis and better prognosis of genetic diseases in high-risk individuals and also for developing targeted therapies in patients with existing genetic disorders [9,10]. Research has been previously directed towards DNA variations located within coding sequences because of their direct effect on the function of the corresponding gene/protein product. Based on evolutionary studies as well as omics and GWAS data, understanding the underlying mechanism by which noncoding DNA variations alter gene function and identification of differentially expressed genes is critical for identifying genetic factors that increase the risk for common complex diseases [10]. Furthermore, developing computational modeling for predicting the impact of genetic and epigenetic factors on disease prognosis and severity will pave the way for the implementation of precision health strategies focusing on prevention and targeted therapy rather than a ‘one pill fits all’ approach [8,9]. 

This Special Issue on DNA Variations in Evolution and Human Diseases features a series of studies that identify new pathological and protective DNA variations in common human diseases and conditions including chronic periodontitis [11], familial adenomatous polyposis [12], asthma [13], rheumatoid arthritis [14], multiple myeloma [15], dental implant failure [16], tooth agenesis [17], tuberculosis [18], and developmental disorders [19,20].
